# Chemical Modification of Silk Fibroin through Serine Amino Acid Residues

**DOI:** 10.3390/ma15134399

**Published:** 2022-06-22

**Authors:** Xiuying Liu, Qianna Xia, Jiao Zhou, Yanbo Zhang, Haiyan Ju, Zhongmin Deng

**Affiliations:** 1 College of Chemistry and Chemical Engineering, Wuhan Textile University, Wuhan 430200, China; liuxiuying@wtu.edu.cn (X.L.); xqn321880456@163.com (Q.X.); zhouj162417@163.com (J.Z.); yanboz@163.com (Y.Z.); 2 Key Laboratory of Advanced Textile Materials and Manufacturing Technology, Ministry of Education, Zhejiang Sci-Tech University, Hangzhou 310018, China; 3 Zhejiang Provincial Key Laboratory of Fiber Materials and Manufacturing Technology, Zhejiang Sci-Tech University, Hangzhou 310018, China; 4 State Key Laboratory of New Textile Materials and Advanced Processing Technologies, Wuhan Textile University, Wuhan 430200, China; 5 Key Laboratory of Textile Fiber and Products, Ministry of Education, Wuhan Textile University, Wuhan 430200, China

**Keywords:** silk fibroin, chemical modification, serine residue, crystalline structure, silk II, β-sheet

## Abstract

Silk fibroin (SF) is a natural protein polymer and promising biomaterial. Chemical modifications have attracted growing interest in expanding SF applications. However, the majority of amino acid residues in SF are non-reactive and most of the reactive ones are in the crystalline region. Herein, a modification was conducted to investigate the possibility of direct modification on the surface of natural SF by a reagent with a mild reactivity, the type and quantity of the residues involved in the reactions, and the structural changes upon modification. Infrared spectrum, ^1^H NMR, titration and amino acid analyses, X-ray diffraction, and hemolysis test were used to analyze the materials. The results showed that sulfonic acid groups were grafted onto SF and the reaction occurred mainly at serine residues through hydroxyl groups. In total, 0.0958 mmol/g of residues participated in the modification with a modification efficiency of 7.6%. Moreover, the crystallinity and the content of β-sheet structure in SF increased upon modification. The modified material had good blood-compatibility. In conclusion, surface modification on native SF through serine residues was practicable and had the advantage of increased β-sheet structure. This will provide an alternative way for the modification of fibroin for the desired application in the biomedical field.

## 1. Introduction

Silk fibers from the *Bombyx mori* silkworm are composed mainly of sericin and silk fibroin (SF). SF, as a natural fibrous protein, is a promising biomaterial for drug delivery matrices, coatings for cell culture, and scaffolds for tissue engineering due to its excellent biocompatibility, robust mechanical properties, and intrinsic biodegradability [1,2,3,4,5,6,7,8,9,10,11]. These properties can be attributed to the unique structure of the protein itself. The primary structure of SF contains highly repetitive hydrophobic regions interspaced with amorphous regions and hydrophilic domains [11,12]. The hydrophobic regions are dominated by repetitive sequences of amino acids, such as glycine (Gly, 42.9~45.9%), alanine (Ala, ~30.3%), serine (Ser, ~12.1%), tyrosine (Tyr, 4.8~5.3%), and valine (Val, ~2.5%) [10,11,12]. The sequences can self-assemble into two secondary structures: silk I (α-helix) and silk II (β-sheets) [12]. Silk I is a metastable crystalline structure and water-soluble [13]. Silk II is highly crystalline and dominates the secondary structure of SF, giving the material robust mechanical properties, resistance to dissolution, and slow biodegradation in vivo [11,12,13]. Due to its structure, the SF macromolecule is insoluble in water and the vast majority of organic solvents [14,15]. The amorphous regions and hydrophilic domains are non-repetitive and contain amino acids which are not found in the hydrophobic region and account for the remaining 4.7%, including threonine (Thr,~0.9%), glutamic (Glu, 0.6~1.4%), aspartic (Asp, 0.5~1.9%), and arginine (Arg, 0.3~0.5%) [12]. In these regions, the random coil conformation is the secondary structure, which was understood as an amorphous, hydrophilic, and less stable structure.

Chemical or physical modifications can alter the structure of SF and enhance the existing physicochemical properties or incorporate new functionalities, expanding the utility of SF protein in a range of biomedical needs [1,6,7,11]. Among the modifications, controlling the content of the β-sheet structure has been studied by many researchers, because the content plays an important role in the material properties including crystallinity, mechanical property, hydrophobicity, silk degradation rates, and diffusion of small molecules [1,11,13]. Current understanding indicates that the crystal silk II (β-sheets) structure can be induced to form by methanol treatment, water annealing, heating, mechanical strain, blending, etc. [13,16]. The crystal silk I can be formed through the addition of polyalcohols with a specific lipophilic-hydrophilic balance [17], aeration of the solution to generate a foam [18], and slow drying with controlled temperature and relative humidity [19].

Besides the modifications through the secondary structure, chemical modifications to exploit the amino acid side chains have also been explored to immobilize functional groups, drug molecules, peptides/enzymes, other polymers, and growth factors on the protein [6,7,11]. These chemical changes reshape important SF properties such as hydrophilicity, immunogenicity, cell adhesion, and differentiation behavior, optimizing silk-based biomaterials for desired needs in the biomedical field. In general, the modification strategies target the reactive residue side chains in SF protein [6,7,11]. However, the majority of amino acid residues in SF are non-reactive, such as Gly, Ala, and Val. However, SF does contain significant quantities of amino acid residues that can be modified, such as Ser and Tyr which are located in the crystalline region [11,12]. 

For modification of silk fibroin, some reactive reagents such as isocyanate, cyanuric chloride, and acid anhydrides had been used in the previous reports. Isocyanates are a family of highly reactive chemicals which are reactive towards not only amino groups but also carboxyl and alcohol/phenolic hydroxyl groups, therefore 3.5 mol% of residues in silk fibroin could participate in the modification using 2-methacryloyloxyethyl isocyanate [20]. Besides the isocyanate, cyanuric chloride is also an effective coupling agent for the attachment of functional molecules to SF [21]. The chlorine atoms from the cyanuric chloride can undergo substitution reactions with the phenolic hydroxyl groups of tyrosine and the amino groups of lysine residues in SF. In addition, acid anhydrides are also highly reactive modification reagents. The anhydride groups were reactive towards not only the free amino groups of the basic amino acid residues and the hydroxyl groups in Ser and Thr but also the phenolic hydroxyl groups in Tyr residues [22,23]. As a result, 41.1–62.7 mol/10^5^ g (0.411–0.627 mmol/g) of acyl and 115–120 mol/10^5^ g (1.15–1.20 mmol/g) of succinyl/glutaryl group were introduced into silk while phthalic/o-sulfobenzoic and succinic/glutaric anhydrides were used for the modification, respectively. 

However, most modification agents have lower reactivity than isocyanate, cyanuric chloride, and anhydrides towards the reaction site of amino acid side chains in SF protein. In literature, the most commonly utilized modification method is derivatization of the carboxylic acid residues via carbodiimide coupling with primary amines [7,11], although SF protein only contains a small number of carboxylic acid residues including Glu (0.6~1.4%) and Asp (0.5~1.9%) [10,11,24]. In order to expand upon the functionalization strategies for SF, Murphy et al. developed a modification method using diazonium coupling chemistry which functionalized the tyrosine residues in SF to control the structure and hydrophilicity of SF protein [24]. The homogeneous modifications were carried out in the regenerated SF aqueous solutions and 20–63% of the Tyr residues were involved in the modification, which tripled the amount of functional group incorporation over carbodiimide coupling methods, as Tyr comprises approximately 5% of the total amino acid content of SF. The method is applicable to aniline derivatives.

The plasma glow technique is an effective modification method and SO_2_ gas plasma treatment had also been applied to introduce sulfonate groups onto silk fibroin film, for the sake of improving the antithrombogenicity of the film [25]. Furthermore, Gu et al. had also applied NH_3_ plasma treatment to produce reactive amino groups on the surface of regenerated silk fibroin film, in order to graft sulfonate groups onto the film through the reaction between the produced amino groups and 1,3-propane sultone [25]. The modification was based on the fact that 1,3-propane sultone is a kind of reagent with a mild reactivity while silk fibroin contains only a small number of residues bearing free amino functional groups including Lys (0.2~0.4%) and Arg (0.3~0.5%) which are more reactive than hydroxyl groups. In the report, the direct modification of silk fibroin by 1,3-propane sultone had not been carried out and the types of amino acid residues that were involved in the modification had not been studied either [25]. 

**Figure 1:**
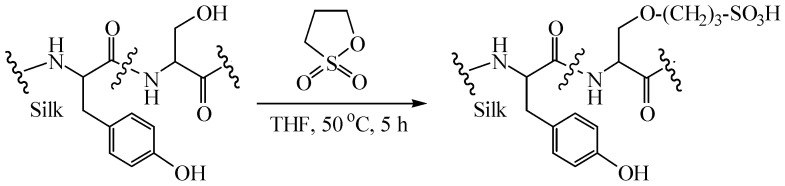
Modification of SF through serine amino acid residues using 1,3-propane sultone.

In this paper, we are interested in the direct surface modification of the native SF fiber targeting the serine residues using a reagent with a mild reactivity without a catalyst, because serines are the most abundant reactive amino acid residues in SF and many commercially available reagents are not as reactive as isocyanate, cyanuric chloride, and anhydrides [11]. A few methods to modify the SF serines which bear the hydroxyl groups have been documented including cyanuric chloride activated coupling [20], enzyme-catalyzed reactions using tyrosinase [26], and sulfation of the serines/tyrosine residues with reactive chlorosulfonic acid and concentrated sulfuric acid [27,28,29]. Herein, 1,3-propane sultone (PS) is selected as the modification reagent based on the consideration that it is less reactive toward the hydroxyl groups than isocyanate, cyanuric chloride, and acid anhydrides. It displays mild reactivity toward the alcohol hydroxyl groups and the reactions were generally catalyzed by a base such as NaOH or sodium alkoxide [30,31]. The reactions between PS and phenolic hydroxyl group required even more alkaline bases such as NaH [32]. Another reason for using PS for the modification was based on the reports that the graft of sulfonate or sulfonic acid groups onto polymers improved the biocompatibility of the polymers [25,33,34,35]. Therefore, the modification effect can be evaluated by testing the biocompatibility of the modified material. In this research, an investigation of the possibility of the reactions between the Ser hydroxyl groups of the natural SF fiber and PS was conducted. The possible reactions are shown in Figure 1. 

Infrared spectrum, ^1^H NMR, titration and amino acid analyses, X-ray diffraction, and hemolysis test were used to analyze the materials to investigate the direct surface modification of the native SF fiber targeting the serine residues. In detail, the modification aimed to investigate whether the functional groups were grafted onto SF, the types of amino acid residues involved in the reactions, the amount of the grafted functional groups onto SF, the modification extent, the blood compatibility of the modified material, and the changes in the crystalline structure of SF caused by modification. The modification was expected to provide an alternative method for the chemical modification of silk fibroin and expand the utility of this family of fibrous proteins in biomedical applications.

## 2. Materials and Methods

### 2.1. Materials

*Bombyx mori* raw silk fibers from China Tongxiang Siyuan Textile Company were degummed in an aqueous solution of 0.02 M Na_2_CO_3_ for 0.5 h. The process was repeated twice and then the fibers were thoroughly rinsed with water for the complete removal of sericin protein. Then SF was dried at 100 °C and ground by a miller to powder. All other reagents were of analytical grade and used without further purification.

### 2.2. Surface Modification of SF

In a three-necked flask which is equipped with a thermometer, magnetic stirrer, and reflux condenser, 0.5 g SF, 20 mL of tetrahydrofuran, and 7.0 mL of 1,3-propane sultone (PS) were mixed and stirred at 50 °C for 5 h. The mixture was subsequently filtered and washed with tetrahydrofuran three times to remove excess PS. The sulfonated SF (SSF) was dried in an oven at 100 °C.

### 2.3. Characterization by FTIR, ^1^H NMR, and UV-Vis

Fourier transform infrared spectra (FTIR) were recorded on a Tensor 27 Bruker FTIR spectrometer. ^1^H nuclear magnetic resonance (^1^H NMR) spectra were recorded on a Bruker advance III digital NMR spectrometer. UV-Vis absorption was measured with a Beijing Puxi TU-1950 spectrophotometer. 

### 2.4. Amino Acid Analyses and Determination of Sulfonic Group Content

The amino acid composition and content were determined using a Hitachi L-8900 amino acid analyzer after acid hydrolysis of a sample with 6 mol/L HCl. The content of the grafted sulfonic acid group was determined through acidimetric titration. Briefly, samples were soaked in 100 mL of deionized water for 0.5 h and then were titrated with 0.01 mol/L sodium hydroxide solution.

### 2.5. X-ray Diffraction (XRD) Analysis

XRD curves were recorded with a Rigaku Ultima IV X-ray diffractometer using Cu Kα radiation (*λ* = 1.54184 Å). Irradiation conditions were 40 kV and 40 mA at a scanning rate of 10° min^−1^. XRD data were analyzed using Software MDI Jade 6 (Materials Data Inc., Livermore, CA, USA). Following background subtraction, individual diffraction peaks were fitted (R < 2%) with pseudo-Voigt profiles (Lorentzian factor set to 0.5). The fitting procedure provided independent estimates of peak position. The crystallinity of the sample was also calculated by the software. 

### 2.6. Hemolytic Assay

The hemolytic assay was performed in the method described in the literature [36], with some modifications. Human whole blood was supplied by healthy donors and diluted with 3.8% sodium citrate at a ratio of 1:9 (*v*/*v*). The sample was incubated with a mixture of 0.5 mL of normal saline and 10 μL diluted blood at 37 °C for 60 min. The mixture was then centrifuged at 3500 rpm for 5 min at room temperature. The absorbance of the supernatant fluid was measured at the wavelength of 540 nm using a Thermo Scientific Varioskan LUX multimode microplate reader. The diluted blood incubated in normal saline and double distilled water was taken as negative and positive control respectively. The hemolytic rate was calculated as:

Hemolysis (%) = (A_s_ − A_N_)/(A_P_ − A_N_) × 100%,

where A_s_, A_P_, and A_N_ denoted the absorbance of sample, positive control, and negative control, respectively.

### 2.7. Statistical Analysis

The data were represented as mean ± standard deviation (SD) with a sample size of 3. Statistical differences between sample pairs were analyzed in Excel using the Student’s *t*-test. Values with *p* < 0.05 were considered statistically significant.

## 3. Results and Discussion

### 3.1. FTIR Spectrum

**Figure 2:**
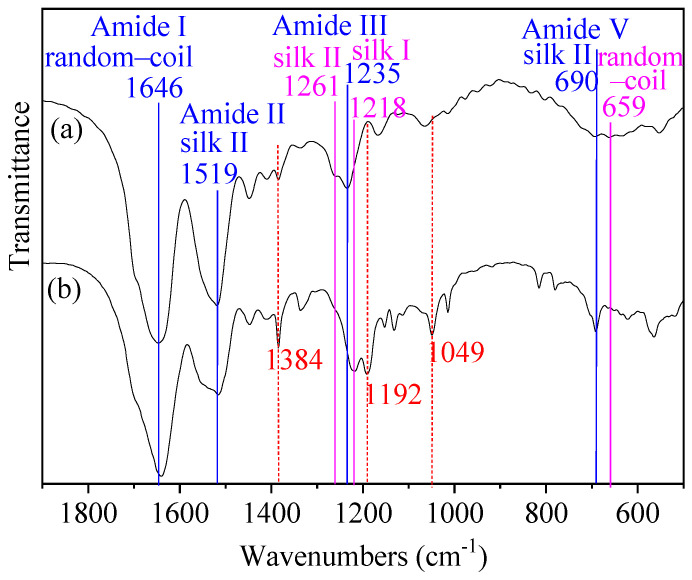
FTIR spectral changes between (**a**) SF and (**b**) SSF reflecting the changes of functional groups and the secondary structures of the protein upon modification.

In order to investigate whether SF was modified by PS, FTIR spectra of SF and SSF were compared (Figure 2). By contrast, SSF exhibited new peaks at 1049 and 1192 cm^−1^ and showed stronger peaks at 1384 cm^−1^. These bands are characteristic absorption of sulfonic groups. These results indicated that the reaction between SF and PS occurred and the functional groups -SO_3_H were grafted onto SF. 

In addition, the FTIR spectra region within 1700–1600, 1600–1500, 1300–1200, and 700–600 cm^−1^ is assigned to the absorption of the peptide backbones of amide I, amide II, amide III, and amide V in SF protein, respectively [19,37,38,39]. The peak positions of the amide bands (amide I, II, III, and V) are known to be sensitive to changes in the secondary structure of SF protein. Therefore, FTIR has been commonly used for the analysis of secondary structures of silk fibroin [19,37,38,39]. As showed in Figure 2, some peaks attributable to absorption of the peptide backbones of SF amides changed upon modification. The first band change was that of amide V at 659 cm^−1^ (random-coil). It nearly disappeared in the spectrum of SSF which only exhibited a peak at 690 cm^−1^ (silk II), revealing a structural transition from amorphous random-coil (non-crystalline structure) to silk II (crystalline structure) [37]. The second change was the band shift of amide III from 1235 cm^−1^ (silk I) to 1218 cm^−1^ (silk I). The third change was the disappearance of the shoulder peak of amide III at 1261 cm^−1^ (silk II), indicating a conformational transition from silk II to silk I upon modification. As to amide I and amide II, SF and SSF exhibited similar structures and displayed bands at about 1646 (random-coil) and 1519 (silk II), respectively [19,38]. In conclusion, FTIR indicated the presence of random-coil form, silk I, and silk II form in both SF and SSF, as well as the structural change from non-crystalline structure to crystalline structure upon modification. 

### 3.2. Structure Characteristics

**Figure 3:**
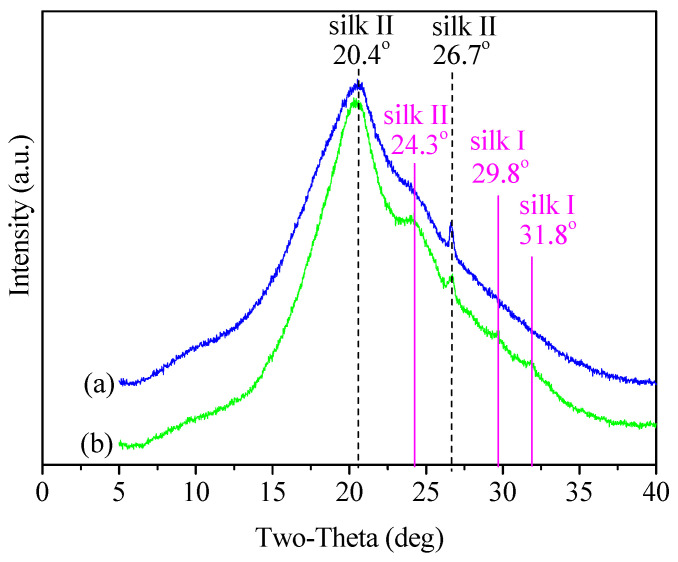
XRD spectra of (**a**) SF and (**b**) SSF.

In order to investigate the changes in the crystalline structures, XRD measurements were performed in detail. As shown in Figure 3, both SF and SSF were characterized by a major and broad diffraction peak at 2θ values of 20.4° and a minor peak at 26.7°, corresponding to silk II crystalline *d* spacing of 4.35 Å and 3.34 Å, respectively [13,40]. These spectral features indicated that both SF and SSF have a prevalent silk II crystalline structure. Besides that, SSF displayed a more obvious peak than SF at 24.3°, corresponding to silk II spacing of 3.66 Å [41]. It revealed a structural transition to silk II form upon modification, consistent with FTIR results which suggested a transition of amide V from random-coil to silk II form. Additionally, SSF showed very minor peaks at 2θ values of 29.8° and 31.8°, corresponding to silk I crystalline spacing of 3.00 Å and 2.81 Å, respectively [19,42]. It exhibited the conformational transition to silk I form, in agreement with the FTIR result about amide III. In summary, XRD results indicated that SSF still had a prevalent silk II crystalline structure and surface modification induced an increased content of crystalline structure. 

**Figure 4:**
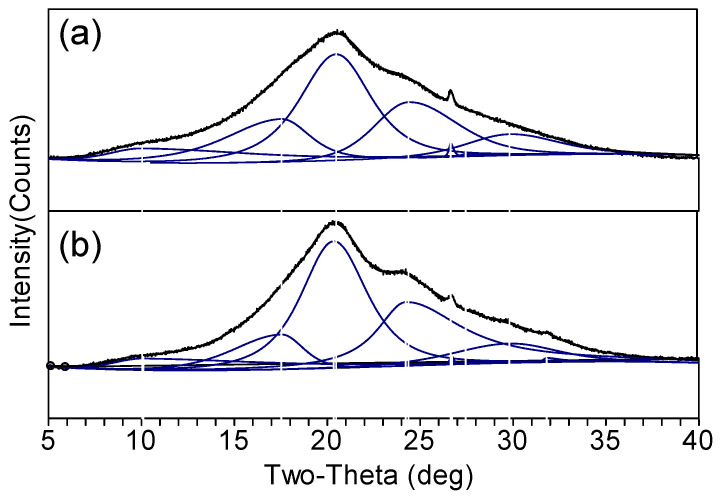
Curve fitting result of XRD spectra of (**a**) SF and (**b**) SSF.

For quantitative interpretation of the structural change between SF and SSF, a deconvolution directly to the XRD spectra was performed as reference data [43], as shown in Figure 4. Deconvolution was performed using Pseudo-Voigt functions with peak positions identified and the area percentage of the component band obtained, as listed in Table 1. The *d* spacings were assigned according to the previous literature [13,19,40,41,42].

The results confirmed that both SF and SSF had prevalent silk II structures, as manifested by the area percentage (relative proportion). For SSF, the relative proportion of silk II structure reached 71.3%. It is worth mentioning that this value was calculated from the XRD spectrum but it was very close to the value (74%) that was evaluated from the ^13^C NMR spectrum for methanol-treated silk fibroin by Asakura and coworkers [37]. For control SF, the proportion of silk II structure (62.4%) was lower. The results indicated that the surface modification increased the content of silk II (β-sheet) structure, as the methanol treatment did [37]. Additionally, the relative proportion of non-crystalline structures in SSF decreased and the silk I structure change little, as compared with SF. These results were consistent with that of FTIR.

In a previous report, Cebe et al. determined the crystallinity value of fibroin films through fast scanning calorimetry analysis [13]. Hu and coworkers evaluated the degree of crystallinity of fibroin films using the deconvolution and curve fitting of Fourier transform infrared spectra [38]. Herein, the crystallinity values of SSF and SF were also studied using Herman’s method from XRD. In the method, the value was calculated using the ratio of the integrated crystalline area to the total diffraction area [44]. As a result, the crystallinity of SSF was 80.7%, higher than that of control SF (71.9%). It was also higher than the regenerated fibroin (59.9%) [44].

### 3.3. ^1^H-NMR Analysis

**Figure 5:**
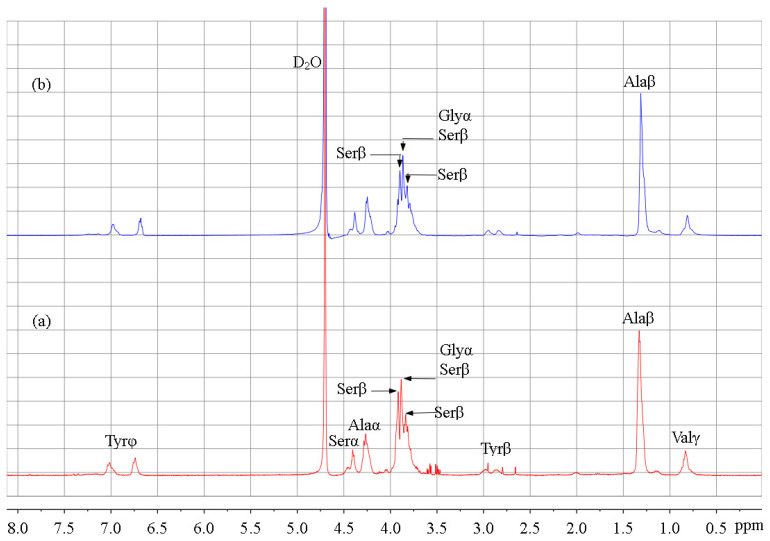
^1^H NMR spectra of (**a**) SF and (**b**) SSF.

In order to determine the reaction site of SF, ^1^H-NMR spectra of SF and SSF were compared (Figure 5). Signals were assigned according to the previous literature [27,28,45]. Some amino acid residues showed no signals in the spectrum due to their low content [28]. The signal change was not obvious because the modification was performed on the surface of solid SF. For this reason, the signal intensity of Ala was used as a reference for comparison, taking into account that Ala bears a methyl side chain and it did not participate in the reaction.

According to the previous report, the signals at 3.82 and 3.92 ppm are attributed to the serine residues [27,28,45]. Upon modification, these signals reduced in intensity, indicating that the modification of SF occurred at Ser residues [27,28]. Ser residues bear hydroxyl groups and are in the crystalline domains of SF [11]. Furthermore, decreased signal intensity was also observed for the peak at 3.88 ppm which was assigned to Ser and Gly residues [45]. Considering that Gly residues have no side chain and bear no reactive groups, the signal changes also indicated that the modification occurred mainly at Ser residues. In conclusion, ^1^H-NMR results revealed the reaction between PS and Ser residues through the hydroxyl group (Figure 1). However, no changes were observed for Tyr residues bearing phenolic hydroxyl group, possibly due to its lower reactivity towards PS. In addition, Thr residues are also hydroxyl side chains that are reactive with PS. Besides that, Lys and Arg are amine side chains and the free amino groups could react with PS [24]. However, these reactions were impossible to confirmed via ^1^H-NMR spectra because the amounts of Thr (~0.9%), Lys (0.2~0.4%), and Arg (0.3~0.5%) residues in silk fibroin are few [10,11,28]. 

### 3.4. UV-Vis Spectrum

**Figure 6:**
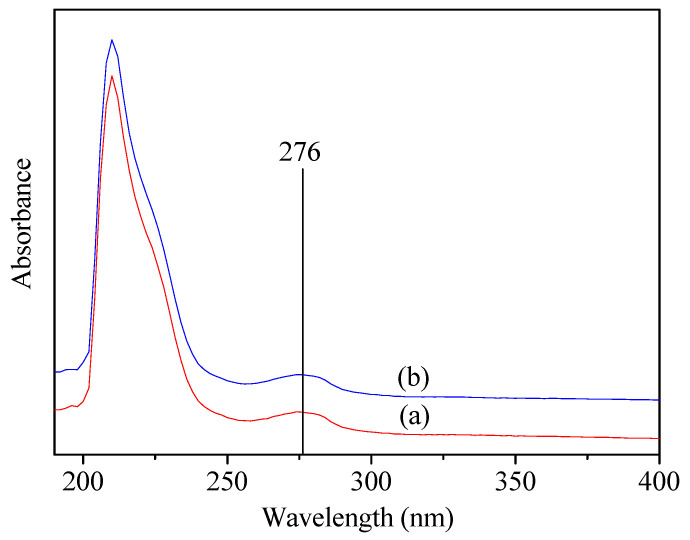
UV-Vis spectra of (**a**) SF and (**b**) SSF.

To further confirm that the Tyr residues were not involved in the reaction, SF and SSF solution with the same concentration was analyzed with UV-Vis spectrum. As shown in Figure 6, both displayed absorption at 276 nm, which was attributed to the absorbance of the Tyr residues [24]. By contrast, no absorption wavelength shift was observed between SF and SSF. In addition, SF and SSF exhibited the same absorbance at 276 nm. Neither wavelength shift nor absorbance decrease indicated that no reaction occurred at tyrosine residues [24], which was consistent with the ^1^H NMR results. According to the above results, Ser residues in SF could be modified by a reagent with a mild reactivity even in the solid-state and without catalyst, but Tyr bearing phenolic hydroxyl groups were not involved in the reaction. This provides researchers with a strategy of SF modification through the most abundant active serine residues in addition to tyrosines and carboxyl-containing residues in low content. 

### 3.5. Optimization of Modification Conditions

**Figure 7:**
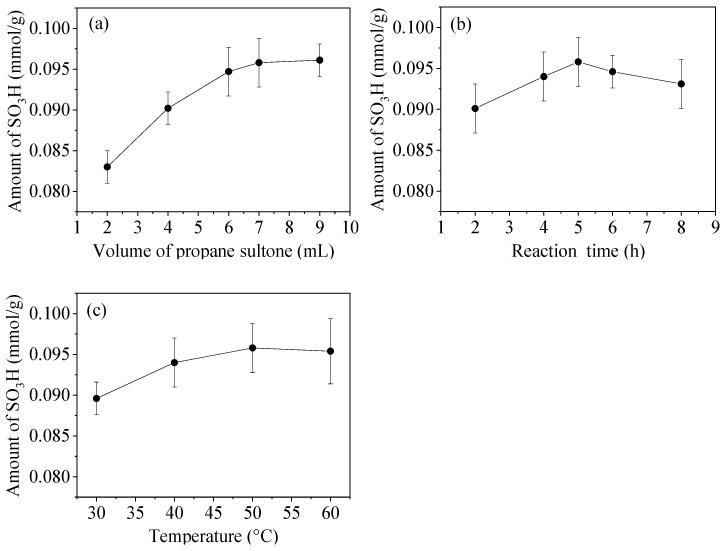
Effects of (**a**) volume of PS, (**b**) reaction time, and (**c**) temperature on the amount of sulfonic acid groups grafted onto SF.

Modification conditions including dosage of PS, reaction time, and temperature were investigated based on their effect on the amount of grafted sulfonic acid groups (Figure 7). 

With the temperature and the duration of the modification process fixed at 50 °C and 5.0 h, respectively, the amount of sulfonic groups per gram of SF increased with an increasing volume of PS (Figure 7a). However, only a slight change was observed while increasing the volume to 9.0 mL, therefore, a volume of 7.0 mL was adopted for further investigation. With the volume of PS and the temperature fixed at 7.0 mL and 50 °C, respectively, the reaction time was prolonged (Figure 7b). The results showed that the surface amount of -SO_3_H per gram of SF was the maximum and reached 0.0958 ± 0.0003 mmol while the reaction time was 5 h. While the volume of PS and the reaction time were fixed at 7.0 mL and 5 h, respectively, the amount of -SO_3_H increased with increasing the temperature from 30 to 50 °C (Figure 7c). The value almost remained unchanged while increasing the temperature to 60 °C. In conclusion, the suitable modification conditions were the reaction temperature of 50 °C, the reaction time of 5 h, and the PS volume of 7.0 mL. Under this condition, sulfonic groups per gram of sample reached 0.0958 ± 0.0003 mmol. 

### 3.6. Modification Extent

Amino acid composition and content of SF were determined (Table 2), in order to calculate the modification extent. The reaction occurred mainly at serine residues according to ^1^H NMR and UV-Vis spectra. Additionally, Thr, Lys, and Arg were also possible residues for reactions. Based on the results in Table 2, the total amount of Ser, Thr, Lys, and Arg residues per gram of SSF was 1.2606 mmol, which is the theoretical amount of sulfonic groups that could be grafted onto SSF in a homogeneously aqueous solution [27]. For the solid SSF, the amount of sulfonic group on the surface reached 0.0958 ± 0.0003 mmol/g, as determined through titration. Therefore, it suggested that 0.0958 ± 0.0003 mmol residues were modified and the modification extent was calculated as 7.6 ± 0.02%. The value was roughly 1/3 to 1/9 of the estimated percent of tyrosines modified in each silk molecule through the diazonium coupling method (20–67%) [24]. However, the molar content of Ser residues was near twice that of Tyr in silk fibroin and the total molar content of Ser, Thr, Lys, and Arg was 2.4 times that of Tyr; therefore, the amount of hydroxyl and amine residues that was modified by PS was about 4/5 to 4/15 of the estimated amount of Tyr modified in each silk molecule through diazonium coupling method. In comparison, the modification extent was low partly because the modification by PS was carried out on the surface of solid SF and the majority of the Ser residues are buried in the hydrophobic domains making them inaccessible for reaction with PS. By contrast, the diazonium coupling was carried out in a homogeneously aqueous SF solution and Tyr residues from both the surface and the inner SF were accessible for reaction. As to the effect of silk fibroin form on the modification efficiency, Freddi and coworkers also reported that about 30% and 10% of the Tyr residues could be oxidized by tyrosinase in regenerated silk fibroin aqueous solutions and in silk gels, respectively, but little or no reaction was detected on silk fibroin powders and fibers [26]. In conclusion, surface modification of native SF through Ser residues was practicable and the modification extent of hydroxyl and amine residues was 7.6 ± 0.02%. 

### 3.7. Hemolysis Percent

Many types of assays are used in blood-compatibility evaluation of materials in the ISO standards [46]. In order to assess the blood compatibility of sulfonated silk fibroin in the form of film, Gu and coworkers used in vitro antithrombogenicity study [25], while Murphy et al. adopted cell culture assays [24]. Ma and coworkers applied the anticoagulant activity and the platelet adhesion [35]. Hemolysis is also a parameter of the blood compatibility of medical materials [36,46]. Here, the hemolysis tests were performed to evaluate the blood compatibility of the samples in the form of powders. The results are shown in Table 3. The positive reference is 100% hemolytic and the negative reference is 0%. In the hemolysis assay, the material was considered to have little or no hemolytic reaction when the hemolytic rate is below 5% [36,47,48]. Silk fibroin exhibited good blood compatibility as a natural biomaterial. A decrease in the hemolytic rate was observed between SF and SSF, which is attributed to the grafted sulfonic groups. The effect of the grafted sulfonic acid groups on the hemolytic property was not as significant as those on cell proliferation/differentiation and antithrombogenicity, probably because the amount of the groups which were grafted onto the surface of solid SF was lower [24,25,35]. However, the hemolytic rates of SSF at both concentrations were below 5% and were both lower than those of SF. The effect of the grafted groups on the decrease in the hemolytic rate was similar to heparin which was immobilized onto silk fibroin as an anticoagulant [36]. The results indicated that SSF had superb biocompatibility.

Compared to the previous studies, the disadvantage of this modification is the lower efficiency of the direct modification on the solid SF surface than the modification in the homogeneous solution of regenerated silk fibroin or on the fibroin surface treated with NH_3_ plasma, which resulted in less improvement of biocompatibility. The advantage of this modification is the relatively high crystallinity and content of the β-sheets structure of the modified SF. Therefore, its potential application is to prepare silk-based materials that require high crystallinity and low biocompatibility. Another potential application of this study is to provide researchers with a strategy of modification through the most abundant active serine residues in addition to tyrosine and the carboxyl groups-bearing residues in low content. It can be expected that the modification efficiency will be higher if the modification is conducted for regenerated silk fibroin in a homogeneous solution, which will lead to improved biocompatibility.

## 4. Conclusions

Chemical modification of native silk fibroin on the solid surface using 1,3-propane sultone, a reagent with mild reactivity, was practicable. Serines were the main amino acid residues that participated in the modification. The modification efficiency of silk fibroin was 7.6 ± 0.02% and 0.0958 ± 0.0003 mmol/g amino acid residues were involved in the modification. The content of the crystal silk II (β-sheet) structure and the crystallinity degree increased upon modification. The modified material was blood compatible. This will provide an alternative method for the modification of silk fibroin and expand the utility of this family of fibrous proteins in biomedical applications.

## Figures and Tables

**Table 1:** Curve-fitting results of SF and SSF.

Peak Position (°)	*d* Spacing (Å)	Structure	Area Percentage (%)		Total Area Percentage of Non-Crystalline/Silk II/Silk I Structures (%)	
			SF	SSF	SF	SSF
10.1	8.76	non-crystalline structures	10.0	8.3	28.1/62.4/9.5	19.3/71.3/9.4
17.5	5.05	non-crystalline structures	18.1	11.0		
20.4	4.35	silk II	39.7	41.8		
24.3	3.66	silk II	22.3	29.3		
26.7	3.34	silk II	0.4	0.2		
27.4	3.25	silk I	0.3	0		
29.8	3.00	silk I	9.2	8.9		
31.8	2.81	silk I	0	0.5		

**Table 2:** Amino acid composition and content of SSF.

Amino Acid	Content (mmol/g ± SD)	Amino Acid	Content (mmol/g ± SD)	Amino Acid	Content (mmol/g ± SD)
Gly	3.9596 ± 0.0986	Glu	0.1708 ± 0.0026	Lys	0.0366 ± 0.0018
Ala	2.7310 ± 0.0138	Thr	0.1048 ± 0.0006	Met	0.0103 ± 0.0002
Ser	1.0549 ± 0.0208	Ile	0.0787 ± 0.0012	His	0.0188 ± 0.0005
Tyr	0.5280 ± 0.0117	Phe	0.0786 ± 0.0016	Arg	0.0643 ± 0.0002
Val	0.2257 ± 0.0017	Leu	0.0772 ± 0.0027	Cys	0
Asp	0.1810 ± 0.0007	Pro	0.0760 ± 0.0036		

**Table 3:** Hemolysis percentage of SF and SSF.

Sample	Concentration (μg/mL)	Hemolytic Rate (% ± SD) (*n* = 3, *p* < 0.05)
Positive control	-	100
Negative control	-	0
SF	100	3.170 ± 0.239
	500	3.275 ± 0.143
SSF	100	2.552 ± 0.195
	500	2.667 ± 0.121
